# Longitudinal Observational Study on Quality of Life in Patients with Chronic Wounds Using DLQI and EQ-5D

**DOI:** 10.3390/medicina61050907

**Published:** 2025-05-17

**Authors:** David Palomar-Albert, Jorge Zamora-Ortiz, Federico Palomar-Llatas, Marta Escudero-Martínez, Alba Naranjo-Cuellar, Maria Isabel Pastor-Orduña

**Affiliations:** 1 Integrity and Skin Care, Integrity and Skin Care Research Group, Catholic University of Valencia SanVicente Mártir, 46001 Valencia, Spain; david.palomar@ucv.es (D.P.-A.); federico.palomar@ucv.es (F.P.-L.); marta.escudero@ucv.es (M.E.-M.); albanaranjo_89@hotmail.com (A.N.-C.); 2 Ulcers Unit, Valencia General Hospital Department, 46014 Valencia, Spain; jorzaor@gmail.com; 3 Doctoral School, Catholic University of Valencia San Vicente Mártir, 46001 Valencia, Spain; 4 Advanced Nursing Unit in Rheumatology and Rehabilitation, Sagunto Hospital, 46500 Sagunto, Spain

**Keywords:** chronic wounds, quality of life, DLQI, EQ-5D, longitudinal study, wound treatment, observational study, patient-centered care, venous ulcers

## Abstract

*Background and Objectives*: Chronic wounds severely impair patients’ quality of life (QoL), impacting physical, emotional, and functional well-being. Understanding the multidimensional effects of treatment is key to implementing effective, patient-centered care strategies. This study aimed to assess changes in QoL among patients with chronic wounds using the Dermatology Life Quality Index (DLQI) and EuroQol-5D (EQ-5D), comparing outcomes across treatment modalities. *Materials and Methods*: A longitudinal observational study was conducted between 2019 and 2024 across three hospitals in the Valencian Community. A total of 278 patients with venous lower-limb ulcers of more than six weeks’ duration were included. Quality-of-life assessments were performed at baseline, one-month follow-up, and discharge. Treatments included alginate, foam, moist wound healing (MWH), compression therapy, and negative-pressure wound therapy (NPWT). Statistical analysis involved Friedman’s test and repeated-measures ANOVA. *Results*: Significant improvements were observed in overall QoL across most treatment modalities. EQ-5D scores progressively increased, while DLQI scores decreased. Pain, embarrassment, and limitations in daily life (e.g., shopping and social activities) showed marked reductions. MWH and foam demonstrated the most favorable impact on QoL, while NPWT showed more modest improvements, possibly due to patient complexity. Notably, the variable “sexuality” remained unchanged (mean = 0.00), possibly due to underreporting or communication barriers. *Conclusions*: Chronic wound treatments significantly improve patients’ quality of life, particularly in terms of pain and social functioning. The use of combined tools (DLQI and EQ-5D) allows for a more comprehensive understanding of these outcomes. These findings highlight the importance of tailoring wound care to individual needs and addressing psychosocial domains, including sexuality. Community nursing, nutritional support, and long-term follow-up should be incorporated into care plans to optimize results, especially in older adults.

## 1. Introduction

Chronic wounds, such as venous leg ulcers, pressure injuries, and diabetic foot ulcers, represent a growing public health problem, especially in developed countries where the population is aging and the prevalence of chronic diseases is rising [1,2]. These wounds are defined as those that fail to proceed through an orderly and timely reparative process to produce anatomical and functional integrity within three months [3].

Chronic wounds have a profound impact not only at the clinical level, due to their morbidity and frequent complications, but also economically, generating substantial healthcare costs related to prolonged treatments, hospitalization, and the need for specialized dressings and devices [4,5]. Moreover, they impose an important psychological and social burden on patients, affecting their quality of life (QoL) across multiple dimensions, including physical function, emotional well-being, autonomy, and social participation [6,7].

Pain, exudate, malodor, and restrictions in mobility are among the main factors that diminish QoL, leading to social isolation, stigmatization, depression, anxiety, and a diminished sense of personal worth [8,9]. In addition, the chronicity of the wounds and the often limited therapeutic success contribute to frustration and emotional exhaustion for both patients and healthcare providers.

Traditionally, the effectiveness of chronic wound management has been evaluated mainly through objective clinical outcomes, such as healing rates, wound size reduction, infection control, and recurrence prevention [10]. However, in recent years, there has been an increasing emphasis on incorporating patient-centered outcomes into the assessment of therapeutic efficacy, recognizing that wound closure alone does not necessarily restore the patient’s overall well-being [11].

The evaluation of QoL in patients with chronic wounds requires the use of validated and sensitive instruments that can capture the multidimensional impact of the disease. Among these, the Dermatology Life Quality Index (DLQI) and the EuroQol-5D (EQ-5D) are two of the most widely employed tools in clinical research and practice [12,13].

Wounds still rely on cross-sectional designs that capture only a static image of the patient’s situation, without considering the temporal evolution of their condition or their progressive adaptation to the disease [14]. This methodological limitation restricts the understanding of how therapeutic interventions actually influence the trajectory of QoL over time.

Longitudinal studies, by contrast, allow for the observation of intra-individual changes, identifying specific moments when deterioration or improvement occurs and relating these to clinical events such as infection, hospitalization, or therapeutic change [15]. Such studies are essential to comprehensively evaluate the effectiveness of wound care strategies and to tailor interventions more precisely to the evolving needs of patients.

Within this framework, it is particularly relevant to analyze not only the physical dimensions of QoL but also those related to social and emotional aspects, including domains that are often neglected in research, such as sexuality and intimate relationships. These areas, although deeply affected by the presence of chronic wounds, are rarely explored due to cultural, emotional, and methodological barriers [16]. The invisibility of these issues perpetuates the suffering of patients even when clinical healing is achieved.

In addition, the influence of chronic wounds on autonomy and activities of daily living is critical, especially in elderly populations, where functional decline is closely linked to health outcomes such as institutionalization and mortality [17]. Therefore, a holistic approach to QoL assessment must include not only symptom control and wound healing but also the preservation of independence and social participation.

Given these considerations, there is an urgent need for longitudinal observational studies that evaluate the QoL of patients with chronic wounds throughout the treatment process, using validated and sensitive instruments capable of capturing both physical and psychosocial dimensions of the disease [18]. Such studies not only contribute to a more comprehensive understanding of patient experiences but also provide critical information for improving clinical practice, guiding therapeutic choices, and optimizing resource allocation.

Furthermore, by including sensitive aspects such as sexuality, functional autonomy, and social reintegration, research can more accurately reflect the true burden of chronic wounds and identify areas where additional support and interventions are required beyond wound healing.

This study aimed to contribute to this field by conducting a longitudinal observational evaluation of QoL in patients with venous leg ulcers, comparing different treatment modalities and exploring psychosocial and functional domains traditionally underrepresented in the literature.

### 1.1. Primary Objective

The primary objective of this study was to evaluate longitudinal changes in quality of life among patients with venous leg ulcers during treatment, using both the Dermatology Life Quality Index (DLQI) and the EuroQol-5D (EQ-5D) instruments.

### 1.2. Secondary Objectives

- To compare the evolution of QoL across different treatment strategies, including moist wound healing (MWH), foam dressings, alginate dressings, compression therapy, and negative-pressure wound therapy (NPWT).
- To explore the specific impacts of chronic wounds on pain, mobility, social participation, autonomy, and sexuality.

## 2. Materials and Methods

This was a longitudinal, prospective, observational, and analytical study conducted between January 2019 and December 2024. The study adhered to the Strengthening the Reporting of Observational Studies in Epidemiology (STROBE) guidelines [1], and the corresponding checklist was submitted as Supplementary Materials.

The research was carried out across three specialized units dedicated to the management of chronic wounds within the Valencian Community, Spain:

- The Ulcer and Wound Unit at the University Clinics of the Catholic University of Valencia (UCV);
- The Advanced Nursing Unit in Rheumatology and Rehabilitation at Sagunto Hospital;
- The Ulcer Unit at the Consorcio Hospital General Universitario de Valencia (CHGUV).

Ethical approval for the study was obtained from the Research Ethics Committee of the Catholic University of Valencia (Reference UCV/2018-2019/080; date of approval: 18 June 2020). The study was conducted in compliance with the Declaration of Helsinki and Spanish legislation on biomedical research involving human subjects. Written informed consent was obtained from all participants prior to enrollment.

### 2.1. Participants and Sample

A consecutive, non-probabilistic sampling method was used to recruit patients attending the three participating centers for evaluation and management of venous leg ulcers. Recruitment took place between January 2019 and December 2023.

The inclusion criteria were as follows:

- Age ≥ 18 years;
- Diagnosis of venous leg ulcer confirmed by clinical evaluation and, when necessary, Doppler ultrasound;
- Ulcer duration greater than six weeks;
- Capacity to provide informed consent;
- Adequate understanding of Spanish to complete the QoL questionnaires.

Exclusion criteria included the following:

- Presence of mixed etiology ulcers (arteriovenous, neuropathic);
- Severe cognitive impairment that precluded reliable questionnaire responses;
- Terminal illnesses or conditions with an expected survival of less than six months;
- Patients unwilling or unable to participate in scheduled follow-ups.

A total of 278 patients met the eligibility criteria and were enrolled in the study. 

### 2.2. Variables and Instruments

The main variables collected included sociodemographic data (age, sex, marital status, employment status, and educational level), clinical characteristics (comorbidities, ulcer duration, location, size, number of ulcers, and presence of infection), and quality-of-life outcomes.

Quality of life was assessed using two validated instruments:

- **Dermatology Life Quality Index (DLQI)**: a specific tool composed of 10 questions grouped into six dimensions (symptoms and feelings, daily activities, leisure, work and school, personal relationships, and treatment) [19]. Each question is scored from 0 to 3, with higher scores indicating greater impairment of quality of life.
- **EuroQol-5D (EQ-5D-5L)**: a generic instrument measuring five health dimensions (mobility, self-care, usual activities, pain/discomfort, and anxiety/depression) on a five-level scale, plus a visual analogue scale (VAS) ranging from 0 (worst imaginable health state) to 100 (best imaginable health state) [20].

Functional independence was assessed using the **Barthel Index**, which evaluates performance in activities of daily living, with a total score ranging from 0 (complete dependence) to 100 (complete independence) [21].

Additional specific variables related to the patient’s subjective experience were collected through an ad hoc structured questionnaire. This included ordinal assessments (0–3 scale) of the impact of the wound on domains such as pain, embarrassment, clothing limitations, social activities, work activities, shopping, sports, sexuality, and emotional state.

Data were collected prospectively at three time points:

- Baseline (Visit 1);
- One-month follow-up (Visit 2);
- Discharge after wound healing or completion of active treatment (Visit 3).

Evaluations were carried out face-to-face by trained healthcare professionals, ensuring uniform administration of the instruments across centers.

### 2.3. Treatment Modalities

Treatment selection was based on clinical judgment, wound characteristics, patient comorbidities, institutional protocols, and the availability of materials at each participating center. The therapeutic approaches included the following:

- **Moist Wound Healing (MWH)**: Application of moist dressings aimed at maintaining an optimal wound environment to promote autolytic debridement and tissue regeneration.
- **Alginate Dressings**: Indicated for wounds with moderate to heavy exudate, providing hemostatic properties and facilitating the removal of devitalized tissue.
- **Foam Dressings**: Polyurethane dressings used for moderate exudate control, cushioning, and protection against external contamination.
- **Compression Therapy**: Essential in the management of venous leg ulcers, employing multilayer systems, elastic or inelastic bandages, or compression stockings according to patient tolerance and clinical indication.
- **Negative-Pressure Wound Therapy (NPWT)**: Applied in complex wounds or in cases requiring enhanced granulation tissue formation and exudate management.

Patients could receive different treatment modalities sequentially or in combination, according to the evolution of the wound. Treatment modifications were documented throughout the follow-up period.

Assignment to treatment groups was observational and non-randomized, reflecting routine clinical practice. This aspect is acknowledged as a potential source of selection bias.

### 2.4. Statistical Analysis

Data analysis was performed using the Statistical Package for the Social Sciences (SPSS) version 25.0 (IBM Corp., Armonk, NY, USA).

Descriptive statistics were calculated for all variables. Categorical variables were expressed as absolute frequencies and percentages, while continuous variables were summarized as means and standard deviations (SDs) or medians and interquartile ranges (IQRs) depending on data distribution.

The normality of continuous variables was assessed using the Kolmogorov–Smirnov test. For comparisons across the three time points (baseline, one-month follow-up, and discharge):

- **Friedman’s test** was applied to ordinal and non-normally distributed variables, followed by Wilcoxon signed-rank tests for post hoc pairwise comparisons with Bonferroni adjustment.
- **Repeated-Measures ANOVA** (with Greenhouse–Geisser correction when sphericity was violated) was used for normally distributed continuous variables.

Only patients who completed all three assessments were included in the longitudinal analysis (complete case analysis).

A *p*-value of <0.05 was considered statistically significant for all tests.

Missing data were analyzed to verify randomness. No imputation methods were employed, as missingness was minimal and judged not to influence the overall results.

### 2.5. Ethical Approval and Informed Consent

The study was approved by the **Ethics Committee of the Catholic University of Valencia** (UCV/2018-2019/080, 18 June 2020). All participants provided informed consent prior to enrollment, and the study complied with the Declaration of Helsinki.

## 3. Results

### 3.1. Baseline Characteristics of the Sample

A total of 278 patients with venous leg ulcers were included in the study. The mean age was 71.4 years (SD: 11.6), with a predominance of females (59.7%). Most participants were retired (68.3%) and had a low educational level (primary education or less: 71.9%).

Regarding clinical characteristics, the mean duration of the ulcer at enrollment was 17.6 months (SD: 14.2), and the average number of ulcers per patient was 1.4 (range: 1–4). Ulcers were predominantly located on the lower third of the leg (82.4%), followed by the malleolar region (13.2%) and other areas (4.4%). Comorbidities were frequent, with hypertension (72.7%), chronic venous insufficiency (69.8%), and type 2 diabetes mellitus (28.7%) being the most prevalent (Table 1).

Active infection at baseline was documented in 23.4% of cases. Compression therapy was being applied at the time of initial assessment in 64.4% of patients. The mean baseline DLQI score was 10.8 (SD: 5.4), indicating a moderate impact on quality of life, and the mean baseline EQ-5D VAS score was 56.7 (SD: 17.5). Baseline functional independence, measured by the Barthel Index, showed a mean score of 78.9 (SD: 17.8), reflecting a moderate degree of dependence.

### 3.2. Evolution of Quality of Life over Time

During follow-up, significant improvements were observed in both specific and generic measures of quality of life.

**Dermatology Life Quality Index (DLQI):**

The mean DLQI score decreased progressively over time:

- Baseline: 10.8 (SD: 5.4);
- One-month follow-up: 7.2 (SD: 4.9);
- Discharge: 4.3 (SD: 4.1).

Friedman’s test revealed a statistically significant difference across the three time points (χ^2^(2) = 178.6; *p* < 0.001). Post hoc analysis confirmed significant differences between all paired comparisons (*p* < 0.001 after Bonferroni correction).

**EuroQol-5D Visual Analogue Scale (EQ-5D VAS)**: EQ-5D VAS scores increased over the study period:

- Baseline: 56.7 (SD: 17.5);
- One-month follow-up: 65.4 (SD: 18.1);
- Discharge: 73.2 (SD: 16.8).

Repeated-measures ANOVA demonstrated a significant effect of time on EQ-5D scores (F (1.8, 498.2) = 156.3; *p* < 0.001), with improvements noted at each follow-up.

### 3.3. Evolution of Specific Psychosocial and Functional Variables

Ordinal variables reflecting specific aspects of patient experience also showed significant improvements:

- **Pain**: Decreased from a mean score of 1.81 to 1.24 (*p* < 0.001).
- **Embarrassment**: Reduced from 1.54 to 0.87 (*p* < 0.001).
- **Clothing limitations**: Slight reduction from 1.18 to 0.95 (*p* = 0.004).
- **Social life impact**: Decreased from 1.47 to 0.82 (*p* < 0.001).
- **Work limitations**: Limited change due to the high number of retired participants.
- **Shopping difficulties**: Decreased significantly (*p* < 0.001).
- **Sexuality domain**: No substantial change observed across time points, with mean scores consistently close to zero.

### 3.4. Evolution of Functional Independence

**Figure 1:**
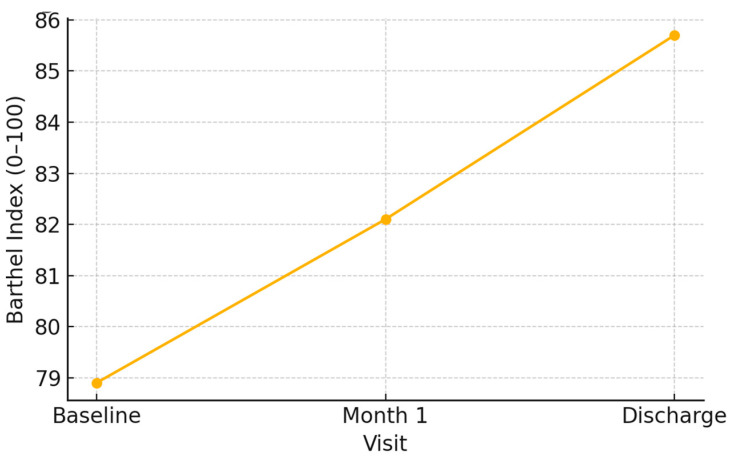
Illustration of the progressive changes in the Barthel Index across the study period, highlighting the evolution of functional independence among patients. While some individuals exhibited improvements in daily activity performance, others showed minimal changes, suggesting that wound healing alone does not fully restore physical autonomy. The data underscore the need for multidisciplinary rehabilitation strategies to enhance mobility and daily function.

The Barthel Index showed heterogeneous trajectories:

- Most patients improved slightly in autonomy, particularly regarding mobility and self-care domains.
- However, a subgroup with multiple comorbidities experienced a decline in independence over the study period (Figure 1).

### 3.5. Comparison of Quality-of-Life Outcomes Across Treatment Modalities

The evolution of quality of life varied depending on the treatment modality applied.

**Figure 2:**
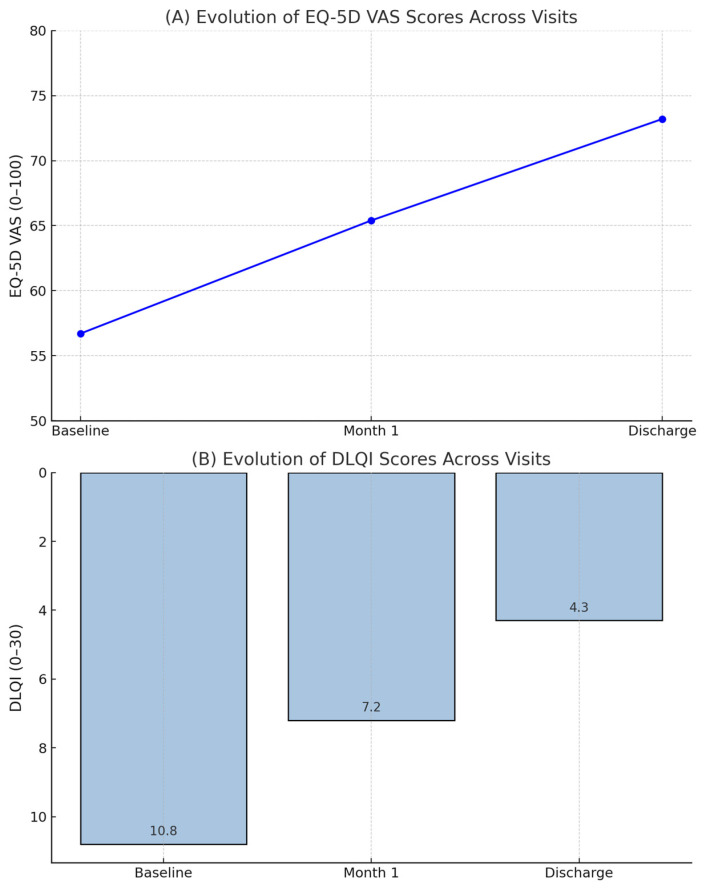
Evolution of health-related quality of life (HRQoL) across study visits. (**A**) Progression of EQ-5D Visual Analogue Scale (VAS) scores at baseline, 1-month follow-up, and discharge. (**B**) Progression of Dermatology Life Quality Index (DLQI) scores across the same visits. Higher EQ-5D VAS scores and lower DLQI scores indicate better perceived quality of life.

1. **Moist Wound Healing (MWH)** and **foam dressings** were associated with the most notable improvements in both DLQI and EQ-5D scores.
2. Patients treated predominantly with MWH achieved a mean DLQI reduction of 7.4 points and an EQ-5D VAS increase of 18.2 points from baseline to discharge (*p* < 0.001 for both comparisons). Foam dressings demonstrated similar outcomes, with slightly lower magnitudes of change.
3. **Alginate dressings** produced moderate improvements in quality of life, particularly in domains related to exudate control and dressing comfort, though overall QoL gains were somewhat smaller compared to MWH and foam dressings.
4. **Compression therapy**, used either as primary treatment or in combination with dressings, resulted in significant improvements in mobility and pain-related dimensions. Patients adhering strictly to compression regimens showed better gains in EQ-5D mobility and pain domains compared to non-adherent patients (*p* = 0.003).
5. **Negative-Pressure Wound Therapy (NPWT)** showed less marked improvement in QoL scores compared to other modalities. While wound healing outcomes were favorable, patients treated with NPWT reported persistent difficulties related to autonomy and social life, likely reflecting the complexity and severity of their underlying wounds (Figure 2).

## 4. Discussion

This longitudinal study confirms that chronic wounds exert a profound impact on patients’ quality of life (QoL) and that structured treatment protocols lead to significant improvements across multiple QoL dimensions.

Both disease-specific (DLQI) and generic (EQ-5D) instruments demonstrated marked improvements from baseline to discharge, reflecting not only clinical healing but also enhancements in physical, emotional, and social well-being. These results are consistent with previous findings that highlight the multidimensional burden of chronic wounds and the potential reversibility of certain impairments through targeted interventions [1,2].

Among the different treatment modalities, moist wound healing (MWH) and foam dressings were associated with the greatest QoL improvements. These therapies optimize the wound environment, enhance patient comfort, and reduce dressing changes, all of which contribute to better perceptions of health and autonomy. The positive role of compression therapy, particularly in mobility and pain control, further supports its fundamental place in the management of venous ulcers, as emphasized in contemporary guidelines [3,4].

In contrast, patients treated with negative-pressure wound therapy (NPWT) experienced less improvement in QoL scores. Although NPWT promotes wound healing in complex cases, its impact on autonomy, body image, and social participation appears limited, likely due to the burden imposed by device management and the severity of the wounds treated. These findings align with other studies indicating that more aggressive or invasive therapies, although clinically effective, may not translate into proportional gains in perceived QoL [5,6].

Pain, embarrassment, and restrictions in social activities were domains that improved significantly during treatment. However, certain dimensions, such as the impact on sexuality and intimate relationships, showed minimal change.

This highlights a persistent gap in the holistic management of patients with chronic wounds: although physical healing progresses, emotional and relational sequelae may persist, often remaining unaddressed in routine clinical practice [7].

### 4.1. Perspectives for Clinical Practice

The results of this study have several implications for clinical practice.

First, they reinforce the importance of incorporating quality-of-life assessments into routine management of patients with chronic wounds. While wound size reduction and healing rates remain essential clinical outcomes, the subjective experience of the patient—including pain control, emotional well-being, autonomy, and social reintegration—must be systematically evaluated to ensure comprehensive care [8].

The combined use of disease-specific and generic QoL instruments, as adopted in this study (DLQI and EQ-5D), provides a more nuanced and complete understanding of patient outcomes. The DLQI captures subtle changes related to symptoms, daily functioning, and social interaction, while the EQ-5D offers a broader evaluation of health-related quality of life and allows comparability with other chronic conditions.

Second, the findings highlight the critical role of treatment modality selection in shaping QoL outcomes.

Therapies such as moist wound healing and foam dressings, which prioritize patient comfort and reduce the logistical burden of care, appear to offer not only clinical efficacy but also superior patient-reported outcomes. The promotion of compression therapy adherence is essential, given its impact on mobility and pain management, particularly in the context of venous leg ulcers.

Third, the persistent impairment observed in sensitive domains such as sexuality calls for greater attention in clinical consultations. Healthcare providers must be trained and encouraged to address these aspects proactively, overcoming cultural taboos and integrating psychosocial support into wound care programs [9].

Finally, nutritional support emerges as a key factor in optimizing wound healing and maintaining functional independence, particularly among elderly patients. Malnutrition is a well-known risk factor for delayed healing and increased morbidity in chronic wound patients. Community nurses play a strategic role in assessing nutritional status, promoting adequate protein and micronutrient intake, and coordinating multidisciplinary interventions to enhance overall recovery [10].

These considerations align with the broader concept of Healthy Aging promoted by the World Health Organization, emphasizing the need to maintain intrinsic capacity and functional ability in older adults, beyond the mere management of specific diseases [11].

### 4.2. Study Limitations

Several limitations must be acknowledged when interpreting the findings of this study.

First, the observational design and the non-randomized allocation to treatment groups may have introduced selection bias. Although treatments were assigned according to clinical judgment and institutional protocols, unmeasured confounding factors could have influenced both the choice of therapy and the outcomes observed.

Second, the exclusive use of complete-case analysis, without imputation for missing data, may limit the generalizability of the findings. However, given the low rate of loss to follow-up, the risk of significant bias is likely minimal.

Third, while the DLQI and EQ-5D instruments are validated and widely used, they may not fully capture certain specific domains particularly relevant to chronic wound patients, such as financial burden, body image disturbances, or caregiver dependency.

Fourth, the sexuality domain was evaluated using a simple ordinal scale rather than a comprehensive instrument. This approach may have underestimated the true burden in this sensitive area, where underreporting is common due to personal and cultural barriers.

Finally, the study population was limited to patients with venous leg ulcers, and the results may not be generalizable to other chronic wound etiologies such as diabetic foot ulcers or pressure injuries.

Future research should focus on expanding the assessment tools to include broader psychosocial and economic dimensions, adopting randomized controlled designs when feasible, and exploring targeted interventions for domains persistently impaired despite clinical healing, such as intimacy, emotional resilience, and functional independence.

## 5. Conclusions

The treatment of chronic venous leg ulcers through structured, patient-centered interventions leads to significant improvements in quality of life across multiple dimensions, beyond mere clinical healing.

The combined use of disease-specific (DLQI) and generic (EQ-5D) instruments enables a comprehensive evaluation of patient outcomes, highlighting both physical and psychosocial recovery.

Treatment modalities that prioritize comfort, autonomy, and adherence, such as moist wound healing and foam dressings, are associated with superior QoL improvements.

However, persistent challenges remain in addressing sensitive domains such as sexuality and emotional resilience, underscoring the need for holistic care approaches.

Integrating quality-of-life assessment, nutritional support, psychosocial counseling, and community-based interventions into routine wound management is essential to optimize outcomes and promote Healthy Aging in this vulnerable population.

Future research should aim to refine QoL measurement tools, develop targeted strategies for domains resistant to improvement, and expand the evidence base.

## Figures and Tables

**Table 1:** Ordinal variables: pain, embarrassment, shopping and clothing, social, sports participation, work, work/social, family, sexuality, and subtraction of time.

Variable	Baseline (Mean ± SD)	Month 1 (Mean ± SD)	Discharge (Mean ± SD)	*p*-Value (Friedman Test)
**Pain (0–3)**	1.81 ± 0.72	1.42 ± 0.65	1.24 ± 0.58	<0.001
**Embarrassment (0–3)**	1.54 ± 0.68	1.02 ± 0.57	0.87 ± 0.49	<0.001
**Clothing limitations (0–3)**	1.18 ± 0.55	1.05 ± 0.51	0.95 ± 0.48	0.004
**Social life impact (0–3)**	1.47 ± 0.66	0.95 ± 0.54	0.82 ± 0.47	<0.001
**Shopping difficulties (0–3)**	1.39 ± 0.61	1.02 ± 0.58	0.83 ± 0.50	<0.001
**Sexuality impact (0–3)**	0.26 ± 0.32	0.23 ± 0.30	0.22 ± 0.29	0.421

Notes: Differences between means are significant (Friedman’s test *p* < 0.001). *Mean* = *arithmetic average*; *SD* = *standard deviation*. Differences between means are significant (Friedman test *p* < 0.001). Differences between means are significant (repeated-measures ANOVA with Greenhouse–Geisser correction, *p* < 0.001). *N indicates the number of patients with complete responses for the variable in question across all three visits*.

## Data Availability

The data presented in this study are available on request from the corresponding author. The data are not publicly available due to privacy or ethical restrictions.

## Supplementary Materials

The following supporting information can be downloaded at: https://www.mdpi.com/article/10.3390/medicina61050907/s1.

## List of Abbreviations

Abbreviation	Full Term
DLQI	Dermatology Life Quality Index
EQ-5D	EuroQol 5-Dimension Questionnaire
NPWT	Negative-Pressure Wound Therapy
CT	Compression Therapy
MWH	Moist Wound Healing
SD	Standard Deviation
VAS	Visual Analogue Scale
UCV	Universidad Católica de Valencia
CHGUV	Consorcio Hospital General Universitario de Valencia
