# Supplementary material for: Longitudinal Observational Study on Quality of Life in Patients with Chronic Wounds Using DLQI and EQ-5D
Source: Medicina (Kaunas). 2025 May 17;61(5):907. doi: 10.3390/medicina61050907 (PMC12113039; doi:10.3390/medicina61050907)
Supplement: Supplementary file 1 [file medicina-61-00907-s001.zip › medicina-3575872-supplementary.pdf]

STROBE CHECK LIST

Nº	STROBE Item	Reported in manuscript	Comment
**1**	Title and Abstract	Title and Abstract	Study design clearly indicated
**2**	Background/Rationale	Introduction (paragraphs 1–3)	Health problem clearly explained
**3**	Objectives	End of Introduction	Main and secondary objectives defined
**4**	Study Design	Methods, Section 2.1	Longitudinal observational design described
**5**	Setting	Methods, Section 2.1	Clinical settings and time frame specified
**6**	Participants	Methods, Section 2.2	Inclusion and exclusion criteria reported
**7**	Variables	Methods, Section 2.3	Definition of main variables provided
**8**	Data Sources/Measurement	Methods, Section 2.3	Measurement instruments detailed (DLQI, EQ-5D)
**9**	Bias	Discussion, Limitations section	Potential sources of bias discussed
**10**	Study Size	Methods, Section 2.2	Sample size explained
**11**	Quantitative Variables	Methods, Section 2.3 and 2.5	Handling of quantitative variables described
**12**	Statistical Methods	Methods, Section 2.5	Statistical analysis detailed
**13**	Participants Flow	Results, Section 3.1	Follow-up losses reported
**14**	Descriptive Data	Results, Table 1	Baseline characteristics presented
**15**	Outcome Data	Results, Sections 3.2–3.5	Main outcomes over time described
**16**	Main Results	Results, Sections 3.2–3.5	Key results analyzed
**17**	Other Analyses	Results, Section 3.5	Comparisons across treatment groups
**18**	Key Results Summary	Discussion, Section 4.1	Summary and interpretation of findings
**19**	Limitations	Discussion, Section 4.2	Methodological limitations addressed
**20**	Interpretation	Discussion, Sections 4.1–4.2	Careful interpretation of results
**21**	Generalisability	Discussion, Limitations	External validity discussed
**22**	Funding	Funding Section	Funding sources declared

von Elm E, Altman DG, Egger M, Pocock SJ, Gøtzsche PC, Vandenbroucke JP; STROBE Initiative.

The Strengthening the Reporting of Observational Studies in Epidemiology (STROBE) statement: guidelines for reporting observational studies.
Lancet. 2007;370(9596):1453–7.
